# Lumbar Ganglioneuroma Presenting With Scoliosis

**DOI:** 10.7759/cureus.16794

**Published:** 2021-07-31

**Authors:** Ravi Gaddipati, Joanna Ma, Samantha Dayawansa, Yuan Shan, Jason H Huang, David Garrett, Rabia Qaiser

**Affiliations:** 1 Neurosurgery, Baylor Scott and White Medical Center - Temple, Temple, USA; 2 Pathology, Baylor Scott and White Medical Center - Temple, Temple, USA

**Keywords:** ganglioneuroma, scoliosis, tumor, spine, approaches

## Abstract

Ganglioneuromas are rare, benign tumors arising from the sympathetic nervous system. The presentation of the tumor is variable and may be associated with scoliosis. Few reports of ganglioneuroma associated with scoliosis­ exist and most involve the thoracic spine. Here, we present a 13-year-old female with scoliosis who was found to have a lumbar ganglioneuroma. The patient was treated with a subtotal resection and lumbar spinal fusion to correct her scoliosis in a single-stage operation. The patient's symptoms and scoliosis markedly improved following treatment without any complications. Additionally, we conducted an up-to-date literature review of ganglioneuromas associated with scoliosis that have been published in the last 20 years. We discuss variations in clinical presentation and surgical approach.

## Introduction

Ganglioneuromas are slow-growing benign tumors that arise from sympathetic ganglia with characteristic neuronal clusters. The tumor is relatively rare, accounting for an estimated 0.1-0.5% of central nervous system tumors [1]. Ganglioneuromas are often present in children and are frequently found in the retroperitoneum or posterior mediastinum [2]. Spinal ganglioneuromas typically present with back pain and less commonly weakness and paresthesias. The tumors are typically treated with resection without chemotherapy or radiotherapy. A study of 30 patients demonstrated an 87% survival rate at 10 years and recurrence in 47% of patients [3].

Currently, few reports exist of ganglioneuroma presenting with scoliosis, with the majority of cases occurring in the thoracic spine. Here, we report a 13-year-old female patient who presented with scoliosis secondary to a lumbar ganglioneuroma. We reviewed similar cases, findings, and discuss the management of lumbar ganglioneuromas coincident with scoliosis.

## Case presentation

**Figure 1:**
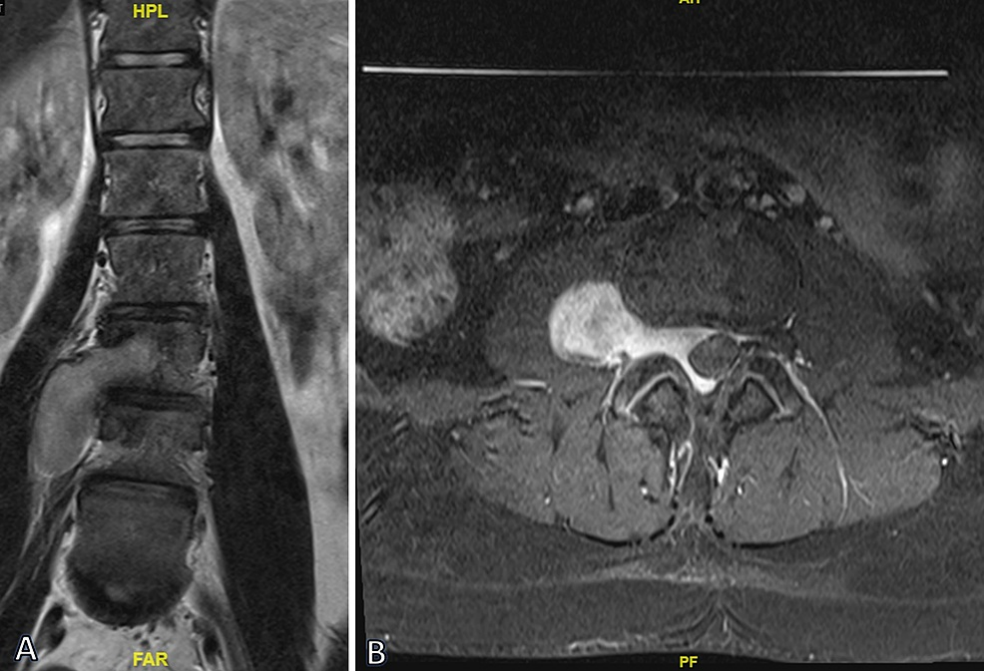
(A) Preoperative coronal T2-weighted MRI demonstrating the mass at the right L3-L4 level with extension into the right psoas muscle and (B) on postcontrast axial MRI with the same mass causing mild effacement of the thecal sac.

A 13-year-old female presented to her primary care physician following a positive scoliosis screening. She reported mild lumbar back pain and intermittent right-sided L3 distribution numbness but denied any paresthesias, weakness, or bowel and bladder symptoms. Physical examination revealed minimal truncal deformity and no neurological deficits. Standing PA and lateral radiographs of the thoracolumbar spine demonstrated a 10-degree right convex curvature of the thoracic spine with the apex at T7 and a 15-degree left convex curvature in the lumbar spine with the apex at L3. Additionally, an osseous lucency was noted at the apex of the lumbar curvature. A subsequent lumbar magnetic resonance imaging (MRI) demonstrated an extrathecal mass measuring 3.8 cm × 2.3 cm in the greatest AP dimension and 4.9 cm craniocaudally (Figure 1). The mass extended through the right L3-L4 neural foramina and into the right psoas muscle. A presumptive diagnosis of schwannoma was made and the decision to wait and observe was discussed with the patient. The patient returned four months later with complaints of 7/10 right leg pain, right L3 distribution numbness, and tingling. A one-stage excision of the lesion followed by an L2-L5 fusion was planned for the patient.

Procedure

**Figure 2:**
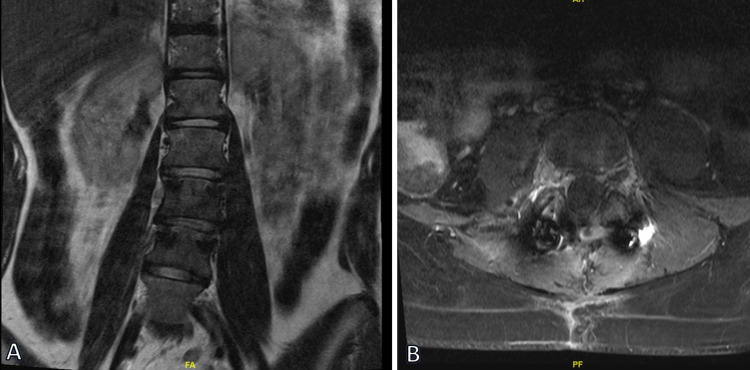
Postoperative MRI demonstrating near-complete excision and fusion of L2-L5. (A) T2-weighted coronal and (B) T1-weighted postcontrast axial MRIs.

**Figure 3:**
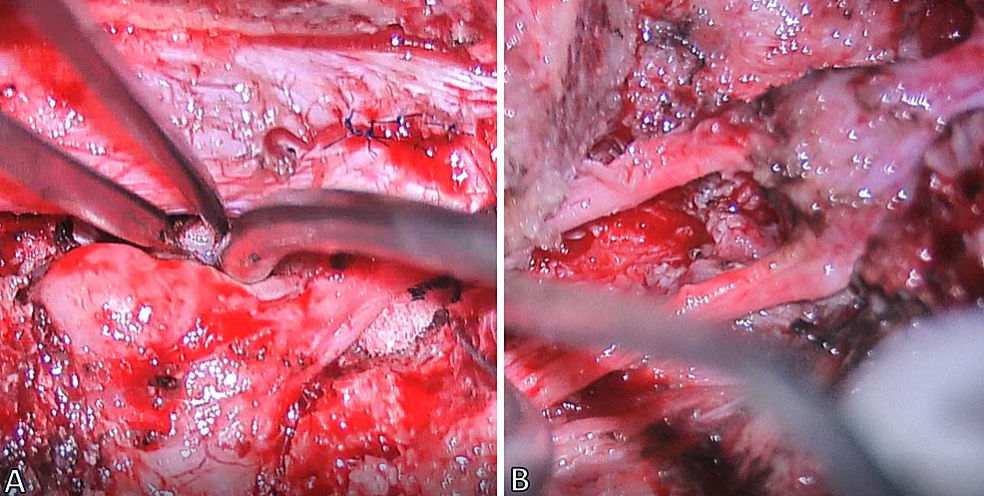
Intraoperative images of the proximal tumor (A) before and (B) after excision.

The procedure was conducted through a posterior approach with the patient in a prone position. The subcutaneous fat and fascia were dissected to expose the transverse processes of L2 through L5. A right L3-L4 laminectomy and subsequent facectomy were performed to provide adequate exposure. Division of the ligamentum flavum revealed the tumor in the vicinity of the right L3 nerve root with extension to the upper right L2 nerve root. The tumor was successfully dissected from the right L3 nerve root with good separation, but it was adherent to the upper part of the right L2 nerve root. A minimal portion was left behind due to the somatosensory evoked potential (SEP) response (Figure 2). Nerve stimulation response after resection was intact following resection (Figure 3).

For the spinal fusion, a Ziehm stereotactic system (Ziehm Imaging GmbH, Nuremberg Germany) was registered to fiducials placed at L1, L2, and L5. A receiving channel was drilled through the pedicles of L2 through L5 and patency was verified with a Lenke probe. Eight pedicle screws were placed to fuse segments L2 through L5. Intraoperative X-ray demonstrated good alignment. The patient was awakened and extubated without complication.

**Figure 4:**
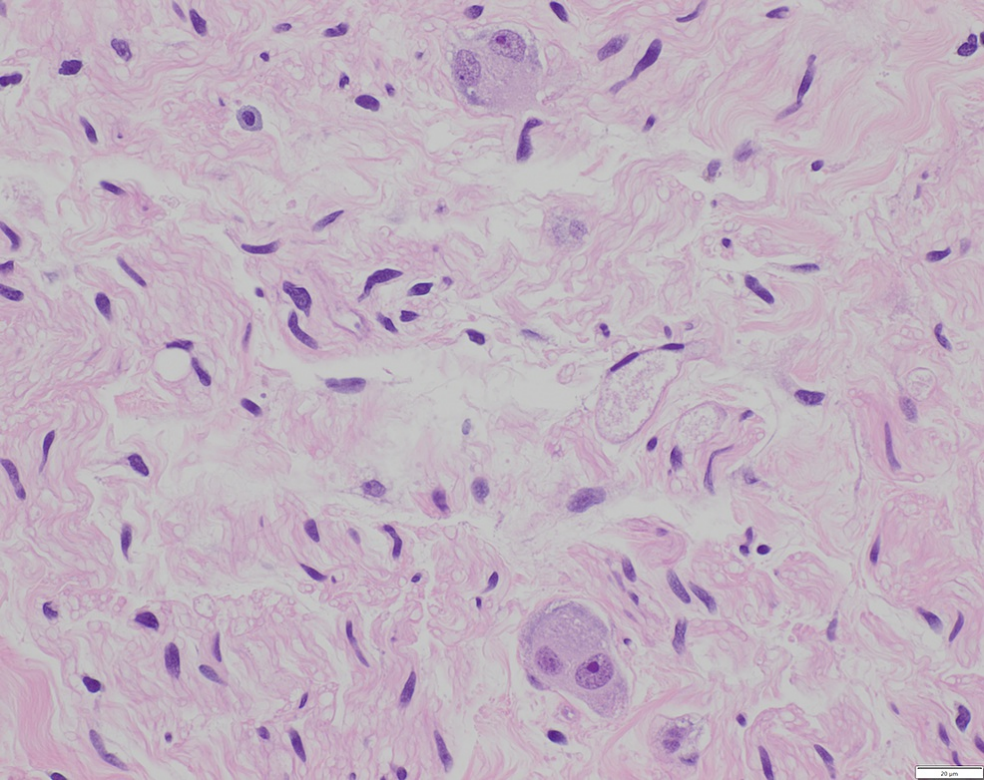
Hematoxylin and eosin stain of the resected mass demonstrating an admixture of Schwann cells and ganglion cells.

Following surgery, the patient was able to ambulate with a walker and reported numbness of the right anterolateral thigh and L3 distribution. The pain and numbness improved over the next two weeks. She continues to do well nearly one year postoperatively and has occasional numbness in the right L3 distribution, but otherwise, her motor examination is normal. The pathology of an intraoperative frozen section demonstrated findings consistent with a schwannoma. Further pathologic evaluation of a second fresh sample confirmed the diagnosis of ganglioneuroma (Figure 4). An Invitae schwannomatosis genetic panel was negative for sequence changes in LZTR1, NF2, and SMARCB1.

Literature review

We conducted a search using PubMed to identify reported cases of scoliosis secondary to ganglioneuroma in the last 20 years (Table 1). Combinations of the keywords “scoliosis,” “ganglioneuroma,” “spine,” and “ganglion,” yielded 17 previously published relevant cases. The median patient age was 16 years (range 7-40). The majority of tumors occurred in the thoracic spine (n = 13), with two occurring in the lumbar spine. Of the 13 cases that reported a follow-up, none had a recurrence at a median follow-up of 24 months. One case was a recurrence from adolescence.

**Table 1:** Literature review of ganglioneuroma presenting with scoliosis in the past 20 years. “Resolution” indicates significant postoperative symptom improvement. RS: resolution of symptoms, NR: no recurrence (months follow-up).

Case	Sex	Age (years)	Scoliosis location	Presentation	Treatment	Outcome
D'Eufemia et al. [4]	F	11	T4-T11	Abdominal pain, nausea, vomiting, constipation	Post. excision	RS, NR (24)
Yang et al. [5]	F	12	T10-L4	Claudication, right abdominal mass, progressive deformity	Two-stage excision and fusion	RS, NR (18)
Qiu et al. [6]	M	9	T9-L1	Asymptomatic	Two-stage excision and fusion	RS, NR (12)
Qiu et al. [6]	F	14	T3-T12	Mild persistent back pain	Two-stage excision and fusion	RS, NR (12), minor loss of correction
Elnady et al. [7]	F	17	T5-T9	Back pain, bilateral lower limb numbness, progressive deformity	Single-stage post excision and fusion	NR (24)
Lopez et al. [8]	M	40	L5-S1	Right lower limb pain, weakness, abnormal gait	Post. excision	11 × 6 mm^2^ residual tumor, pain
Yilmaz et al. [9]	F	10	T11-L5	Backpain, tingling	Post. excision	Symptoms persisted
Algazwi et al. [10]	F	18	T3-T7	Asymptomatic	Excision	RS, NR (72), stable scoliosis
Wang et al. [11]	F	21	Lumbar retroperitoneal	Asymptomatic	Excision	RS, NR (19), stable scoliosis
Takebayashi et al. [12]	M	33	L1-L2	Urinary retention left lower limb numbness	Two-stage extradural and intradural excision	RS
Ulusoy et al. [13]	F	7	T12-L2	Left hypoesthesia at T12, L1, L2 dermatomes	-	-
Zou et al. [14]	F	18	T7-T9	None reported	Ant. excision	RS
Kara and Oztunali [15]	M	28	T1-T7	Dyspnea, vomiting. history of ganglioneuroma	-	Recurrence, RS
Spiegel et al. [16]	F	14	T5-T7	None	Two-stage excision and fusion	RS, NR (24)
Velyvis et al. [17]	F	15	T2-T7	Mild persistent back pain	Ant. excision and post. decompression	RS, NR (72)
Lai et al. [18]	F	12	T8-T11	Asymptomatic	Two-stage excision and fusion	Partial scoliosis correction, NR (24)
This case	F	13	L3-L4	Back pain, scoliosis	Single-stage excision and fusion	RS

## Discussion

Ganglioneuromas of the spine are rare, well-differentiated tumors with variable clinical presentations. The relationship between ganglioneuroma and scoliosis is unclear, limited by the paucity of cases reported. They are sometimes associated with scoliosis which may be the presenting symptom as in this case. The tumor is an important consideration in the differential for idiopathic scoliosis, especially in the context of progressive back pain and neuropathic symptoms. In one case, D’Eufemia et al. reported a misdiagnosed case of scoliosis that was later determined to be secondary with an undiscovered ganglioneuroma [4].

The most common presenting symptom was back pain or a positive scoliosis screening. Asymptomatic presentations were also common. The slowly enlarging tumor often results in progressive symptoms, necessitating complete excision. The management of the co-presenting scoliosis is variable. Yang et al. advocated for separation of excision and spinal fusion into two stages with a one- to seven-week interval in between to enable a shorter operation time [5]. Of the 18 cases reviewed, five patients were treated with this approach. The outcomes were favorable - all resulted in diminished symptoms and no recurrence at the time of follow up. Qiu et al. reported one case of scoliosis progression after fusion [6].

In this case, management of the ganglioneuroma involved near gross total resection and correction of the scoliosis with spinal fusion in a single operation. We found only one other case where a single-stage approach was used [7]. Both resulted in favorable outcomes. Interestingly, many cases opted not to surgically correct the scoliosis (n=9). Of these, seven patients had full resolution of symptoms at the time of follow-up. The cause of residual symptoms may be attributed to residual tumor in one case [8]. The persistence in the case reported by Yilmaz et al. is more unclear, although uncorrected scoliosis may play a role [9].

## Conclusions

Management of ganglioneuroma coincident with scoliosis is variable. In this case, we report favorable outcomes with a single-stage excision of a lumbar ganglioneuroma and lumbar fusion. A literature review revealed a two-stage operation - a common approach that also resulted in favorable outcomes. The decision to correct scoliosis depends on the extent, complexity, and symptoms attributable to scoliosis. This review shows that excision, with or without fusion, is successful in resolving present symptoms and preventing scoliosis progression. More data are needed to determine whether surgical scoliosis correction at the time of excision is more favorable compared to a two-stage approach or excision only.
